# The open access juggernaut and another bastion falls

**DOI:** 10.1308/rcsann.2024.0055

**Published:** 2024-05-30

**Authors:** D Batura

**Affiliations:** London North West University Healthcare NHS Trust, UK

I read the November editorial decision to make the *Annals* an open access (OA) publication with a sense of inevitability.^1^ The pace at which scholarly publications race towards adopting an OA model meant it was only a matter of time before the *Annals* joined the bandwagon.

Much has been written about the advantages of OA publication, offering authors a bewildering array of choices ranging from green, gold and other parts of the colour palette. Proponents of the OA model inevitably extol the advantages of the availability of research for those who lack the resources to purchase access to the subscription model. This is undoubtedly a laudable motive; however, researchers in most parts of the world access paywalled articles through their libraries, national initiatives or academic associations at a fraction of the cost of paying for OA publication; hence, the benefits are moot at best. Furthermore, the oft-used carrot dangled before researchers that OA research garners more citations is fallacious.^2,3^

In the global euphoria for OA publication, it is easy to lose sight of the disadvantages. Imposing OA requirements on funded research risks alienating scholars and ignores the implications of the financial involvement of corporations in academic research.^4^ Because OA fees are not regulated, these can be very high, making self-payment difficult and effectively discouraging research participation from authors who cannot pay.^5^ The only other alternative for authors to publish OA requires those authors who are not funded by their universities or organisations to solicit funds from various public institutions, private corporations and even pharma, raising substantial ethical considerations. Other significant disadvantages include concerns around quality, sustainability and offering a breeding ground for predatory publishing practices. Although most of these do not apply to the *Annals*, a reputed and admired journal for many decades, the article-processing charges (APC) cannot be swept under the carpet. Though the *Annals* will waive the APC for fellows and members of the Royal College of Surgeons of England, to authors based in Hinari Group A countries, and offer a 50% waiver to authors from Hinari Group B countries, those not so affiliated must pay. This would affect a large part of the very constituency that is expected to benefit from a paywall waiver. Those researchers who are not from these groupings or who are not College members and are without recourse to institutional or public funding will find themselves disfranchised and voiceless. Sadly, plus ça change, plus c’est la même chose.
